# Acute-on-Chronic Liver Failure: Pathophysiological Mechanisms and Management

**DOI:** 10.3389/fmed.2021.752875

**Published:** 2021-11-08

**Authors:** Arshi Khanam, Shyam Kottilil

**Affiliations:** Division of Clinical Care and Research, Institute of Human Virology, University of Maryland School of Medicine, Baltimore, MD, United States

**Keywords:** acute-on-chronic liver failure, cirrhosis, immunopathology, liver transplantation, stem cell therapy

## Abstract

Acute-on-chronic liver failure (ACLF) is a multifaceted condition with poor treatment options and high short-term mortality. ACLF can develop in patients with or without liver cirrhosis, where patients with decompensated cirrhosis display a higher risk of short-term mortality. Pathophysiological mechanisms include systemic inflammation due to bacterial and fungal infections and acute hepatic insult with drug, alcohol, and viral hepatitis. Cryptogenic factors also contribute to the development of ACLF. The clinical outcome of patients with ACLF gets further complicated by the occurrence of variceal hemorrhage, hepatorenal syndrome, hepatic encephalopathy, and systemic immune dysfunction. Regardless of the better understanding of pathophysiological mechanisms, no specific and definitive treatment is available except for liver transplantation. The recent approach of regenerative medicine using mesenchymal stem cells (MSCs) could be advantageous for the treatment of ACLF as these cells can downregulate inflammatory response by inducing antiinflammatory events and prevent hepatic damage and fibrosis by inhibiting hepatic stellate cell activation and collagen synthesis. Moreover, MSCs are involved in tissue repair by the process of liver regeneration. Considering the broad therapeutic potential of MSCs, it can serve as an alternative treatment to liver transplant in the near future, if promising results are achieved.

## Introduction

**Figure 1:**
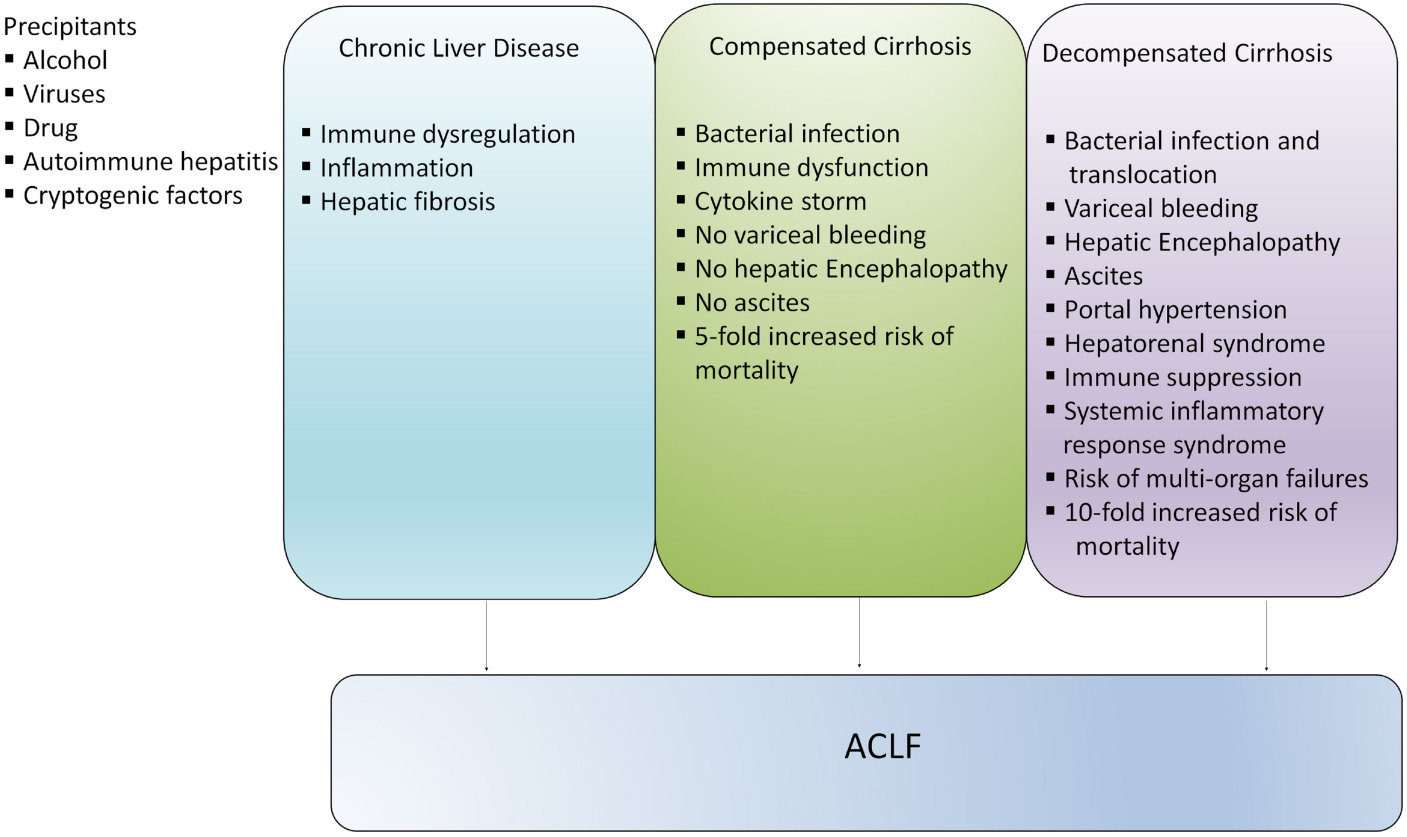
Development of ACLF in different stages of liver disease. ACLF can develop directly in patients with chronic liver disease (CLD), compensated and decompensated cirrhosis or it may progress slowly from CLD to compensated and decompensated cirrhosis and eventually develop into ACLF. However, development of ACLF in patients with compensated and decompensated cirrhosis inflict higher risk of mortality.

Acute-on-chronic liver failure (ACLF) is a serious condition which develops in patients with chronic liver disease (CLD) with compensated and decompensated cirrhosis. ACLF is defined as acute hepatic decompensation, development of multiorgan failure, and high risk of short-term mortality (1–3). Based on different diagnostic criteria, various international consortiums around the world projected distinct definitions for this syndrome. Until now more than 13 different definitions of ACLF have been proposed, but the definitions of the Asian Pacific Association for the Study of the Liver (APASL) ACLF Research Consortium and the European Association for the Study of the Liver-Chronic Liver Failure (EASL-CLIF) Consortium are widely acknowledged (1, 2). There is heterogeneity present between different definitions related to the underlying CLD, precipitating events, and multi-organ failure, though, different definitions provide their consensus over high short-term mortality. Later, it was suggested that the differences in definitions are associated with discrete epidemiology of liver diseases in the Eastern and Western Hemispheres. Recently, North America Consortium for the Study of End-Stage Liver Disease (NACSELD) introduced another definition of ACLF, which defines ACLF as a condition that develops in CLD patients with or without cirrhosis. NACSELD definition also agreed that with high short-term mortality in these patients in the absence of the proper management of underlying liver disease, liver support, and liver transplantation (4) studies are focusing on the further validation of the current definition of ACLF. Management of the underlying cause of CLD with suitable therapies, including antivirals for hepatitis B (HBV)- and hepatitis C virus (HCV)-related liver disease, alcohol abstinence in alcoholic liver disease (ALD), and immunosuppressive therapies in autoimmune liver disease may avoid or reverse the development of cirrhosis (5–7). Contrarily, if the underlying cause is left untreated or it persists in patients with compensated cirrhosis, extended hepatic necrosis can destroy hepatic architecture, increase intrahepatic resistance, portal hypertension, damage liver parenchymal cells, and subsequently cause acute decompensation of the disease (8–10). Since, ACLF may establish at any phase of the disease from CLD to compensated to early or late decompensated cirrhosis, it is not considered as a terminal incidence of long-standing decompensated cirrhosis (11), although the risk of mortality is significantly higher in patients with compensated and decompensated cirrhosis in comparison with the general population (12) (Figure 1). In the present review, we will focus on the discrete pathophysiological mechanisms, complications, and management of patients with ACLF.

## Pathophysiological Mechanisms in ACLF

### Systemic Inflammation

Current advancements in the understanding of the pathophysiological basis suggest that hyperreactive systemic inflammatory response is a critical driver of tissue damage and organ injury in patients with acutely decompensated cirrhosis leading to the development of ACLF (13). Extensive production of inflammatory mediators including cytokines, chemokines, growth factors, bioactive lipid mediators, and expression of chemokine receptors by different immune cells induce systemic inflammation, immune-mediated tissue damage, and subsequently liver failure (14–19). Activated immune cells release other mediators such as proteases, reactive oxygen species (ROS), prostaglandins, and leukotrienes that further aggravate tissue damage (16, 20).

Systemic inflammation may occur in the presence and absence of identifiable and non-identifiable triggers. Identifiable triggers may include bacterial infections, excessive alcohol consumption, and relapse of chronic viral hepatitis, whereas non-identifiable triggers do not have any clinically identifiable cause (21–23). The mechanism of inflammation is not very well-characterized. It is believed that bacterial products and endogenous molecules are potential inducers of inflammation. Patients with acute decompensated cirrhosis and ACLF may develop systemic inflammation even in the absence of bacterial infections and their translocation through the release of damage-associated molecular patterns (DAMPs) from injured tissues and organs (24, 25). Various components of injured or dying cells including cytosol, mitochondria, and nucleus release DAMPs by the process of necrotic, pyroapoptotic, and necroapoptotic cell death which contribute to inflammation (26, 27). Cell death mechanisms including apoptotic pathways influence the recovery of HBV-related ACLF (28). Recently, it has been identified that caspase-cleaved keratin-18 (ck18) can predict the progression of acute decompensation to ACLF (29). Biomarkers including caspase-cleaved neoepitope of cytokeratin-18 and intact cytokeratin-18 variant recognized as M30 and M65, respectively have been investigated in ACLF. A higher ratio of M30:M65 in patients with ACLF can serve as a good indicator of apoptosis severity (30).

**Figure 2:**
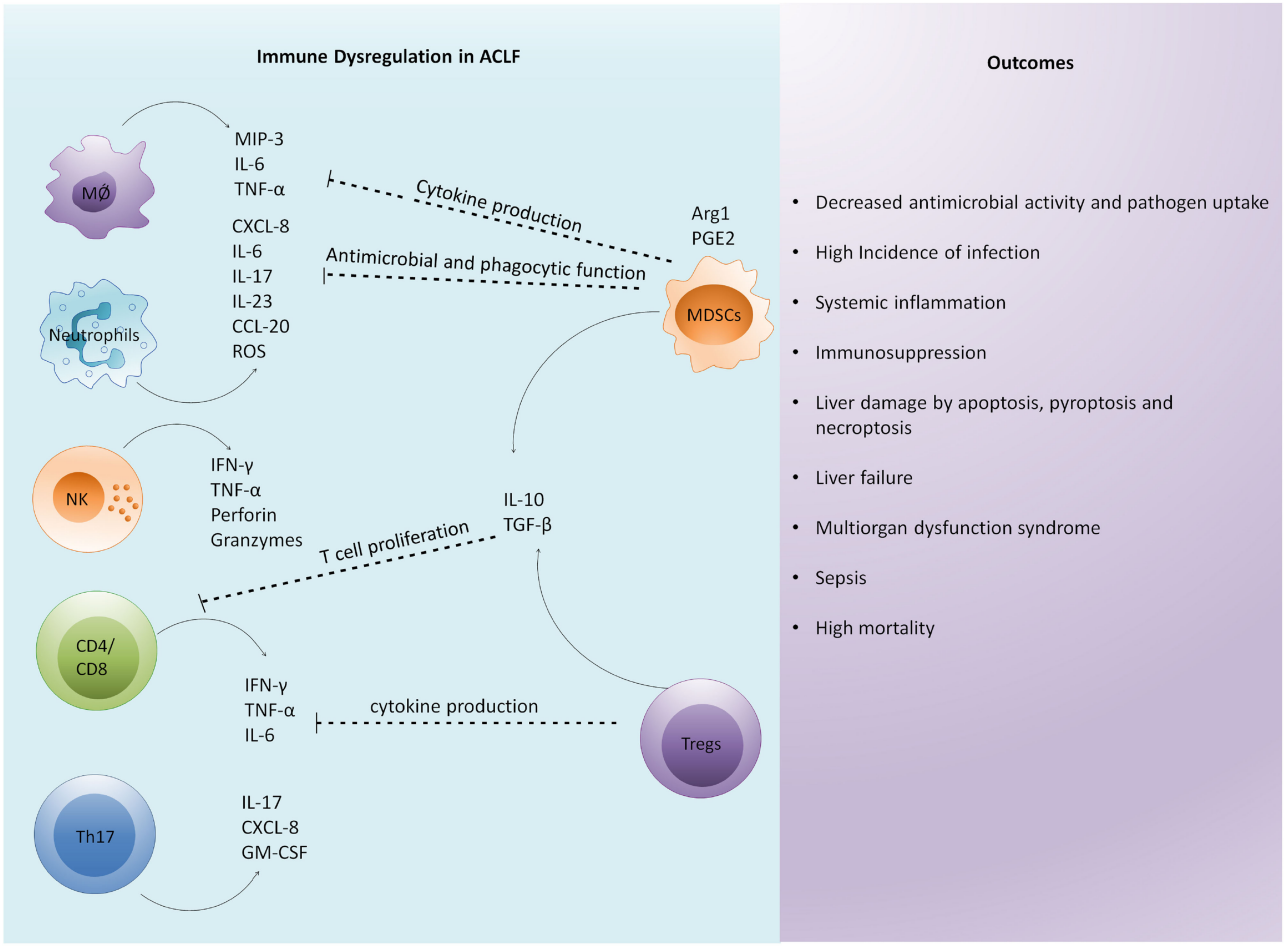
Immune dysregulation is a critical factor in the pathophysiology of ACLF. Excessive immune activation drives systemic and intrahepatic cytokine storms in patients with ACLF leading to inflammation. Enormous cytokine secretion in the liver results in the infiltration of circulating immune cells that further induce hepatic damage. Subsequently, an antiinflammatory response is generated to control excessive inflammation. However, excessive antiinflammatory response by regulatory cells impairs the function of other immune cells by inhibiting their antimicrobial and phagocytic activities, cytokine secretion, and T cell proliferation. The overall immune dysregulation increases the risk of infection and sepsis development. In addition, it induces distinct cell death related pathways in the liver, causing multiorgan dysfunction leading to high mortality. MØ, macrophage; NK cells, natural killer cells; MIP-3, macrophage inflammatory protein; TNF-α, tumor necrosis factor- α; IFN-γ, interferon-γ; ROS, reactive oxygen species; GM-CSF, granulocyte macrophage colony stimulating factor; Arg1, arginase-1; PGE2, prostaglandin E2; MDSC, myeloid derived suppressor cells; Tregs, regulatory T cells; TGF-β, transforming growth factor-β.

Host immune and genetic factors exaggerate systemic inflammation. Different immune cells including monocytes, macrophages, neutrophils, natural killer cells (NK cells), myeloid-derived suppressor cells (MDSCs), CD4, CD8, and Th17 cells immensely contribute to cytokine and chemokine production (16, 31–36) leading to cytokine storm, systemic inflammation, and cell death (Figure 2). In fact, various immune surface molecules comprising chemokine receptors and coinhibitory molecules derive inflammatory responses leading to necrotic and apoptotic cell death and further aggravate hepatic damage (16, 37, 38). Similarly, host genetic factors, for instance, single nucleotide variants might modulate the release of inflammatory mediators by innate immune cells and can change the expression of pattern recognition receptors (PRRs). Genetic variations in genes coding for innate immune receptors including nucleotide-binding oligomerization domain (NOD)-2, mannan-binding lectin (MBL), and MBL-associated serine protease (MASP)-2 are associated with increased short-term mortality in ACLF and patients with acute decompensation (39). In addition, single nucleotide polymorphism with IL-1 gene clusters plays a protective role in patients with acute decompensated cirrhosis by controlling systemic inflammation and reducing the development of ACLF (40).

### Immune Cell Paralysis and Immunosuppression

Immunosuppression also acts as a potential contributor to the pathogenesis of ACLF mainly through the amplification of immune paresis that further increases the risk of bacterial infections (41). MER receptor tyrosine kinase (MERTK) expression on monocytes and macrophages are known to suppress innate immune cells. MERTK expressing monocytes and macrophages were increased in the circulation, liver, and lymph nodes of patients with ACLF and correlated with severity of hepatic and extrahepatic systemic inflammatory response and disease (42). MERTK expressing monocytes exhibited decreased response toward lipopolysaccharide (LPS), and its blockade with UNC569 restored monocytes function (42). Patients with ACLF do not only have a hyperactive inflammatory response, but also hyper antiinflammatory response and dysfunctional immune response exist in parallel. Interestingly, hyperinflammatory and immunosuppressive conditions both coexist in the same individual. However, the prevalence of one or the other depends on sequential and longitudinal aspects. Circulating and intrahepatic immune cells may act differently. Circulating immune cells might display inflammatory phenotype whereas hepatic immune cells may exhibit antiinflammatory phenotype since the liver is an immunotolerogenic organ (38, 43).

An intense antiinflammatory response along with immune dysregulation and exhaustion are associated with immune cell paralysis (44). Inhibitory pathways also exist to maintain immune homeostasis and avoid the overactivation of immune cells; however, hyper-reactive inhibitory pathways cause immune exhaustion and paresis. High expression of programmed death-1 (PD-1) and T-cell immunoglobulin and mucin domain 3 (TIM3) play a crucial role in the immune paresis in patients with alcoholic hepatitis (45). Immunosuppression in alcoholic hepatitis is associated with the pathogenesis of ACLF. Patients with ACLF show a similar degree of cellular immune depression as in severe sepsis that contributes to increased infectious morbidity in these patients (46). Also, patients with ACLF display highly compromised tumor necrosis factor-α (TNF-α) production and HLA-DR expression under *ex vivo* conditions (46). Moreover, immune dysfunction in ACLF is independent of the underlying etiology of liver cirrhosis and is common in all patients. Increased level of prostaglandin E2 (PGE2), an immunosuppressive lipid mediator, inhibits TLR4 expression. PGE2 also inhibits macrophage proinflammatory cytokines in response to LPS; thus, it decreases macrophage bacterial killing in ACLF (47). Expansion of CD14+HLA-DR- MDSCs in the circulation of ACLF decreases T cell proliferation, TNF-α production following TLR stimulation, and has reduced phagocytic potential against *E. Coli* (48). Since MDSCs can impair both innate and adaptive responses to microbial products, it enhances the risk of infections and displays great pathological significance. Immunosuppression also contributes to acquire nosocomial infection in ACLF (49). Generally, it is speculated that immunosuppression is a regulatory mechanism to control the exaggerated inflammatory response; however, there is no proof of the concept, and future studies are required to determine whether the development of systemic inflammation and immunosuppression associate with each other during ACLF.

### Extensive Alcoholism

A high percentage of ACLF cases develop due to excessive alcohol consumption leading to severe alcoholic hepatitis (sAH) and further development of ACLF (50). CANONIC study reported that 25% of ACLF cases occur due to sAH (1). Alcohol not only impairs immune responses but also enables gut bacterial translocation that initiates inflammation (51). One of the most compelling direct effects of alcohol is that it affects the structure and integrity of the gastrointestinal (GI) system as this is the first point of contact for alcohol. Alcohol alters the numbers and relative plethora of gut microbiome affecting the normal gut function, maturation, and function of the immune system, further disturbing the crosstalk between gut organisms and immune system (52). Moreover, alcohol ingestion destructs epithelial cells, activates neutrophils, and T cells, upsetting gut barrier function, resulting in the leakage of microbes into the circulation (50). Disturbance of gut barrier function has critical consequences beyond the intestinal system. Leakage of bacterial products such as LPS from the gut activates the innate immune system in the liver, prompting inflammation and eventually causing liver cirrhosis and cancer (53). Irrespective of whether the bacteria from leaky gut causes infection or not, they release pattern-associated molecular patterns (PAMPs) including LPS that reach the liver and get recognized by toll-like receptors (TLRs) present on hepatic Kupffer cells (KCs), and encourage the production of proinflammatory cytokines and chemokines that chemoattract neutrophils (54). It is well-documented that acetaldehyde metabolism induces ROS. Abundance of ROS causes mitochondrial DNA stress that generates inflammatory response and subsequently contributes to liver failure (55). Moreover, sAH hampers liver regeneration despite the presence of activated hepatic progenitor cells (HPCs) that fail to differentiate into hepatocytes and hence, no replacement of damaged hepatocytes occurs (56). In addition, cumulative effects of alcohol on both innate and adaptive immunity tremendously weaken the host defense, which in turn increases the susceptibility of chronic drinkers toward various infections that further exaggerate systemic inflammation. In fact, it has been reported that alcohol exposure restricts the development of the immune system in the fetus, shown by *in utero* exposure of alcohol. This exposure escalates the risk of a newborn of getting an infection (57). Also, the harmful effects of alcohol on the development of the immune system last into adulthood. Collectively, these findings propose that sAH might be the result of both impaired hepatocyte regeneration and immunopathology.

### Viral Infections

#### HBV Reactivation

Development of ACLF is attributed to both viral and host factors. HBV viral factors include its genotypes, hepatitis B e antigen (HBeAg) status, and mutations in the HBV precore and core promoter regions (58). High viral replication of certain variants has been associated with a more hostile disease course. A strong correlation between HBV DNA level and the development of cirrhosis and HCC has been reported in patients infected with chronic HBV (59).

Hepatitis B virus reactivation is one of the most common precipitating events associated with acute decompensation or ACLF in patients with HBV related cirrhosis (60). HBV reactivation is a well-characterized condition, marked by an abrupt reappearance or rise of HBV DNA in patients with previously inactive or resolved HBV infection. Reactivation is often spontaneous, but can also be triggered by immune suppression or alterations in immune function and after cancer chemotherapy (61). However, the main challenge in diagnosing reactivation of CHB is to differentiate it from acute hepatitis B due to the lack of pathological evidence. A low titer of anti-HBc IgM and high HBV DNA are useful in identifying severe acute reactivation of HBV from acute HBV (62). Also, the presence of basal core promoter mutation and precore stop codon mutations can differentiate severe acute exacerbations of chronic HBV from acute HBV infection (63). Moreover, submissive hepatic necrosis helps in distinguishing HBV-related ACLF from cirrhotic patients with acute decompensation (64). Patients with submissive hepatic necrosis display severely compromised hepatic function, high occurrence of multiorgan failure, and a smaller interval between acute decompensation and liver transplantation. Patients with ACLF with hepatic precipitants, such as HBV reactivation, have short-term mortality similar to patients with extrahepatic precipitant, suggesting that short-term mortality is not related to the presence and type of precipitating events (65). Rather it is the number of organ failures that are related to high mortality and not the etiology of cirrhosis or precipitating events.

Recently, plasminogen, an inactive precursor of plasmin, a potent serine protease that is involved in the dissolution of fibrin blood clots, served as a promising prognostic biomarker for HBV-related ACLF (66). The study reported that P5 is a high-performance prognostic score for HBV-related ACLF and it causes a decrease in plasminogen level at admission associated with mortality. Longitudinal analysis reveals a gradual increase in plasminogen in HBV-related ACLF survivors, but a steady decline in non-survivors. The changes in plasminogen levels imitated the course of improvement, fluctuation, and deterioration. Of note, plasminogen levels were negatively associated with the number of failed organs and were lower in patients with cerebral and coagulation failure, suggesting plasminogen as an independent prognostic factor.

#### Hepatitis E Virus Infection

Hepatitis E virus (HEV) infection is one of the commonest causes of acute viral hepatitis (AVH) around the globe, especially in Asia and Africa, where it causes the epidemic of AVH (67). HEV infection is mostly transmitted through the feco-oral route, and the clinical manifestations differ between patients ranging from asymptomatic infection to uncomplicated AVH and severe fulminant liver failure. Generally, AVH, owing to HEV infection, is an acute and self-limiting illness; however, when it occurs in CLD patients, it may progress rapidly to ACLF resulting in high mortality. Several studies reported HEV infection as one of the main causes for decompensation of cirrhosis in Asia and Africa, where HEV is an endemic. In almost 21% cases of ACLF, HEV infection was the precipitating cause for liver decompensation accounting for 0–67% mortality (67). Though it differs strikingly from the Western countries where HEV infection is hardly the main cause of acute decompensation in ACLF, a Chinese study reported that out of 188 patients with CHB, 136 encountered superinfections with HEV and only 52 patients had hepatitis A virus (HAV) superinfection. Also, complications, liver failure, and mortality were frequent in HEV infected groups, indicating HEV superinfection causes more severe liver disease and poor prognosis than those with HAV superinfection (68). Another study from India reported that 61% of ACLF cases had HEV infection as the main precipitant event, 33% had HAV, and 6% had both the infections (69). Since there is no recommended vaccine against HEV, appropriate precautions such as ingestion of boiled water and well-cooked food in the HEV-endemic regions are required to avoid HEV superinfection. Moreover, ribavirin can be used for the treatment of acute and chronic hepatitis E (70) and can decrease the severity of the disease in patients with acute and chronic liver failure. Though the optimization of the dose and duration is critical as treatment failure may occur, a course of 3 months is the optimal duration for the ribavirin monotherapy, and longer treatment periods are available for the patients with severely compromised immune function (71).

### Autoimmune Hepatitis

Autoimmune hepatitis (AIH) in an infrequent condition but associated with liver disease-related morbidity and mortality. AIH is an immune-mediated, inflammatory condition of the liver, which occurs when the host immune system turns against the liver cells. It is characterized by the presence of circulating autoantibodies, hypergammaglobulinemia, and discrete features on liver biopsy (72) that mainly present necroinflammatory liver disease. It may also include discrete disease subtypes ranging from benign chronic hepatitis and indolent disease to fulminant hepatic failure. Nearly, 20% of patients with AIH present severe jaundice, coagulopathy, and encephalopathy with or without ascites coinciding with the features of ALF or ACLF (73). Clinically it is difficult to distinguish autoimmune-ALF from ACLF (74); though histological features are distinct (74, 75). In AIH-related ACLF, advanced fibrosis, ductular reactions, and huge parenchymal collapse with lymphoplasmacytic inflammation are common, whereas lymphoid aggregates and perivenulitis are less frequent (74). Since AIH is considered uncommon in the Asian Pacific region, AIH flare as a cause of ACLF is frequently disregarded leading to disease severity, delay in treatment, and poor outcome. Treatment options for AIH-related ACLF includes the use of corticosteroids, which demonstrated survival benefits compared with those who did not receive it; although, patients with high MELD score >27 and HE in advanced fibrosis (>−F3) displayed poor corticosteroid response, serving them as predictors of an unfavorable response (74). As AIH is a rare disorder, data is quite limited in this field requiring further investigations.

## Complications in ACLF

### Bleeding

Acute variceal bleeding is a serious complication of liver cirrhosis resulting from portal hypertension. Variceal bleeding is often associated with ACLF and accounts for 70% of all upper gastrointestinal bleeding episodes in cirrhosis (76). Patients with ACLF display an imbalance in systemic and hepatic hemodynamics with severe portal hypertension and worsening of systemic vasodilation (77). The increased portal pressure arises as a consequence of hepatic and systemic inflammation, reduced hepatic perfusion, and high intrahepatic resistance. As patients with ACLF have high baseline hepatic venous pressure gradient and lower hepatic blood flow, the chances of variceal bleeding are also high (78). Although a significant progress has been made in the treatment of acute variceal bleeding comprising transjugular intrahepatic portosystemic shunt (TIPS) (79), endoscopic treatment, and drug therapy, 10–20% of the patients experience treatment failure that associate with a high short-term risk of further liver decompensation and death (80). Recently, a study reported the prevalence of ACLF in patients with acute variceal bleeding and found its association with rebleeding and mortality (79). ACLF nearly doubled the risk of rebleeding and emerged as an independent risk factor for rebleeding and mortality in acute variceal bleeding patients. Patients with ACLF with variceal bleeding may benefit from the placement of TIPS. In fact, the insertion of TIPS improves the 42-day and the 1-year survival in patients with ACLF (79). Also, preemptive placement of TIPS is helpful in patients with ACLF with acute variceal rebleeding.

### Hepatic Encephalopathy

Hepatic encephalopathy (HE) is another frequent manifestation of ACLF. Localized and systemic alterations on the background of cirrhosis are accountable for the pathogenesis of encephalopathy. However, the exact pathophysiological mechanism of HE is not well-defined. Patients with HE manifest a range of neuropsychiatric symptoms including sensory abnormalities, psychomotor dysfunction, and impaired memory (81). Hyperammonemia, systemic inflammation including sepsis, bacterial translocation, insulin resistance, and oxidative stress remain as key factors in the development of HE, driving cerebral edema and inflammation (82). Since, patients with chronic liver failure frequently undergo immunoparesis, an association between ammonia and inflammation has been anticipated. During liver failure, reduced usage of ammonia as a substrate in the ammonia detoxification pathway (urea cycle) and portosystemic shunting increases ammonia accumulation in the systemic circulation (83). Also, impaired hepatic metabolism leads to decreased elimination of nitrogen-based waste products such as ammonia which crosses the blood–brain barrier, where it combines with glutamate to form glutamine (84). Cerebral accumulation of glutamine employs an osmotic effect that leads to increased retention of water in the brain, resulting in swelling and cytotoxic edema. HE in a hospitalized cirrhotic patient is related to high mortality that further increases in case of patients with ACLF (82). The effect of the systemic inflammatory response on ammonia-induced neurological dysfunction has been defined in cirrhotic patients hospitalized with an infection. In fact, systemic inflammation in patients with progressive HE is associated with mortality (85). Existence of severe HE in cirrhotic patients requires management in the ICU, and patients frequently require tracheal intubation for airway protection (86). Careful sedation is also needed. For the precise management of HE, the early step is to identify and reverse any precipitating event such as infection or bleeding (87). Therapies lowering ammonia are commonly used. Moreover, lactulose, a non-absorbable disaccharide that converts into short-chain fatty acids by the colonic microbiome generates an acidic environment, leading to the inactivation of ammonia-producing colonic bacteria, and the conversion of ammonia to non-absorbable ammonium (88). Antibiotics are also recommended (89), generally in combination with lactulose that is helpful in reducing mortality and the length of hospital stay in comparison with lactulose alone (90). As ammonia is considered a key participant in the pathogenesis of HE, antibiotics that reduce the ammonia-producing enteric bacteria including vancomycin, neomycin, paromomycin, and metronidazole are used in combination with or without lactulose (89). These antibiotics serve as second-line agents; though, few of the antibiotics are not acclaimed for long-term use due to nephrotoxicity, ototoxicity, and neurotoxicity. For instance, neomycin is ototoxic and nephrotoxic, whereas metronidazole has neurotoxic effects. Another antibiotic Rifaximin, a nominally absorbed oral antimicrobial agent, is highly efficacious in treating HE through eliminating ammonia-producing colonic bacteria, resulting in reduced ammonia concentration. Rifaximin is poorly absorbed and has minimal systemic bioavailability which favors its long-term use than the other antibiotics (91).

### Concomitant Infection

#### Bacterial Infection

Patients with ACLF are prone to develop infection. Bacterial infections play an essential role in the development and further progression of ACLF, and participate either as a key precipitating event or as a complication (92). At the time of ACLF diagnosis, approximately 37% of the patients exhibit bacterial infections, whereas 46% of the remaining patients with ACLF develop bacterial infections during the 4 weeks follow-up (93). Both Gram-positive and Gram-negative bacteria contribute to the infection (21). The incidence of Gram-positive bacterial infections mainly *Staphylococcus* are increasing than the Gram-negative bacterial infections. The common Gram-positive bacteria includes *Staphylococcus aureus* and *Enterococcus* (94). *S. aureus* causes respiratory tract and also skin infection, whereas *Enterococcus* frequently causes urinary tract infection. Besides, due to the improper use of antibiotics, antimicrobial resistance including methicillin-resistant *S. aureus (MRSA) and Vancomycin-resistance Enterococcus (VRE)* is also increasing in patients with cirrhosis (95, 96). The common Gram-negative bacteria causing infections include *Escherichia coli and Klebsiella pneumoniae* (97). *E. coli* instigate spontaneous bacterial peritonitis (SBP), whereas *Klebsiella pneumoniae* is a common cause of pneumonia. Another Gram-negative bacteria Acinetobacter causes respiratory tract infection. In ACLF, urinary tract, and also skin infections, pneumonia, and SBP are predominant and complicate the condition of these patients (98). In fact, the severity of ACLF measured by the prevalence of organ failure and mortality was higher in patients where ACLF was caused by an infection in comparison to those with non-infectious etiologies (99).

Patients with bacterial infections exhibited a higher grade of systemic inflammation, worse clinical course, and lower probability of 90-day survival than those without infection (100). Cirrhotic patients, especially the decompensated ones, are extremely susceptible to develop bacterial infection due to impaired gastrointestinal barriers and increased gut permeability that allows bacterial translocation to the surrounding tissues and end up in the blood stream leading to systemic inflammation, sepsis, and ACLF development (101). Continuous translocation of bacteria and its products stimulate the immune cells after identification by pathogen-recognition receptors, typically TLRs, causing overwhelming inflammatory response *via* producing inflammatory cytokines (25). High levels of circulatory proinflammatory cytokines induce systemic inflammation and increase disease severity. Further, systemic inflammatory responses encourage organ damage through oxidative stress, endothelial dysfunction, and reduced organ perfusion. Also, pathogen and pathogen-derived endotoxins are efficient in promoting direct tissue damage (49). In general, hepatocytes are moderately protected against LPS-induced tissue damage *via* the induction of NF-κB pathway; however, this mechanism is impaired in cirrhotic patients causing direct tissue damage (102, 103). Moreover, translocation of bacteria or their PAMPs impair the contractility of mesenteric vessels that supply blood to the small and large intestines and increase portal hypertension in cirrhotic patients, which further affect the microbiota and increase bacterial translocation (104). Studies believe that liver, intestinal barrier and microbiota, and immune response preserve equilibrium through complex interactions, and perturbation in this balance leads to increased gut permeability, although the precise mechanism is not clear.

Early diagnosis and appropriate antibiotic use on time are critical factors to improve the prognosis of patients with bacterial infections. Also, biomarkers of infection may aid in the early diagnosis of infection. Acute-phase proteins including C-reactive protein (CRP) and procalcitonin (PCT) are early markers of infection that are frequently used to diagnose the infection (49). A study described that a value of CRP >12.15 mg/L is a good indicator of bacterial infection in patients with ACLF (105). Unfortunately, due to the increased use of antimicrobial agents, antimicrobial resistance has increased over the years. In fact, a study advocated against the use of antibiotics except in distinct conditions such as gastrointestinal bleeding, history of SBP, and ascites fluid protein concentration <1.5 g/dL (95). However, that cannot be considered in patients with septic shock, as each hour delay in antibiotic treatment following identified hypotension can decrease patient's survival up to 7.6% for the first 6 h (106). Due to the prolonged wait time in getting bacterial culture results, we lose vital time to treat the patient with antibiotics; however, antibiotic treatment without identifying the infection will put unwarranted stress on the liver; therefore, techniques that could identify the infections in short span of time are highly needed.

#### Fungal Infection

Persistent impaired immune response and hepatocyte damage reduce the efficiency of inhibiting and clearing the pathogen in ACLF. A study reported the occurrence of invasive fungal disease in 43% of patients with ACLF and observed higher mortality in these patients than those without the invasive fungal disease (107). *Candida* as well as *Aspergillus* species are the common infections in ACLF and primarily infect urinary and respiratory tracts (108, 109). Like a bacterial infection, a fungal infection could act as the main precipitating event in ACLF; however, the mechanism is not well-recognized. It is believed that the exacerbation of ACLF induces immune paralysis, which can lead to invasive fungal infection (108). In addition, the invasive fungal disease is also responsible for increased inflammatory cytokine response that further augments organ failure (107). To identify the fungal infection, specific tests including fungal culture, serologies, and fungal tissue staining are required (108). The invasive fungal infection is diagnosed by 1,3-β-D-glucan and galactomannan index (110). Recently, it has been shown that bacterial and fungal infections are associated with poor clinical course and high 28 and 90-day mortality (111). Fungal infections not only increase short-term (28 days) and medium term (90 days) mortality, but also enhance the risk of 1-year mortality. This finding allows identifying patients with ACLF who survive an infection but are intended for a poor long-term prognosis; hence, close monitoring and specific management is demanding in these patients. The identification of an infection at the initial stage is the most challenging. The current approach is to culture the patient's sample, which is a time-consuming process and inclined to crosscontamination. Therefore, it is critical to identify the infection based on the other clinical markers. Currently, the most common indicators of infection are systemic inflammatory response syndrome (SIRS), PCT, serum lactate, and few others (95, 112). Although few studies support the relation between SIRS and infection in liver disease patients without sepsis, others question the sensitivity of SIRS in critically ill and cirrhotic patients.

## Strategies for the Management of ACLF

**Figure 3:**
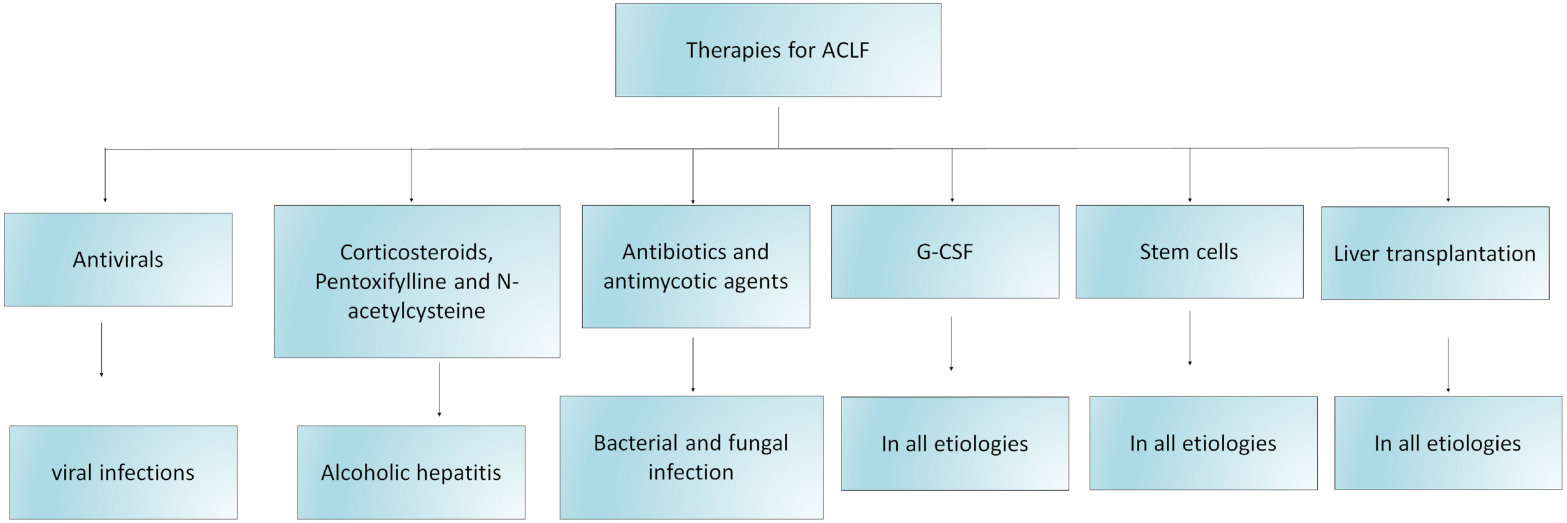
Schematic diagram indicating therapeutic strategies for the management of ACLF. The figure demonstrates different treatment options for patients with ACLF based on their etiology, precipitating events, complications and the requirement of liver transplantation. Several clinical trials are investigating the efficacy of stem cell therapy as one of the potential therapeutic approach for the treatment of ACLF. G-CSF: granulocyte macrophage colony stimulating factor.

At present, there is no specific treatment accessible for patients with ACLF. The management of ACLF includes etiology-based treatment, controlling and treating complications, providing artificial liver support system, and liver transplantation, summarized in Figure 3 and further detailed below. Admission of patients with ACLF should be considered preferably in transplant centers. Organ functions need to be monitored closely and organ-specific treatment is required to restrict the development of multiorgan failures.

### Antivirals for the Treatment of HBV-Related ACLF

The primary aim to use antiviral for the treatment of HBV-related ACLF is to substantially decrease the viral load, thereby inhibiting hepatocyte cell death and improving survival outcomes by constraining the decompensation-related multiorgan complications. Low HBV DNA level at baseline and a further reduction in viral load improve the outcomes in HBV-related ACLF, suggesting that early implementation of antiviral therapy could improve the prognosis of HBV-related patients with ACLF (113). A two log reduction in HBV DNA at 2 weeks improves survival benefits in HBV-related ACLF (114). Antiviral therapy also increases the chances of stabilization to liver transplant time and expands the transplant outcomes. Antivirals including Lamivudine and Entecavir (ETV) displayed short-term survival benefits in HBV-related patients with ACLF, despite the prevalence of drug resistance with Lamivudine (115). Recently, a study demonstrated the role of Tenofovir Disoproxil Fumarate (TDF) and ETV for the treatment of HBV-related ACLF and reported that the short-term efficacy of TDF is greater than ETV (115). Furthermore, TDF showed a higher reduction in HBV DNA level and undetected HBV-DNA in 2 weeks and lowered the model of end stage liver disease (MELD) as well as Child-Turcotte-Pugh (CTP) scores that are potential markers of disease severity. Importantly, the survival rate was higher in patients who received TDF than those who were treated with ETV. HBV-related patients with ACLF treated with Tenofovir Alafenamide (TAF), TDF, and ETV have comparable 48-weeks liver transplant-free survival (116). TAF have similar efficacy in HBV DNA reduction and liver biochemical responses as observed in TDF and ETV group. TAF, TDF, and ETV possess equal safety and efficacy in short as well as long-term treatment of HBV-related ACLF. Several studies demonstrated that ETV had comparable short-term efficacy with LAM; however, more promising in long-term (117). Due to the high incidence of drug resistance, clinical guidelines do not recommend the use LAM, whereas ETV has lower rates of resistance; therefore, widely used in clinical practice. In fact, antiviral treatment using ETV, TDF, and Telbivudine (LDT) is being given to pregnant females who encounter ACLF. These antivirals showed >2 log reduction in HBV DNA levels. A study by Yang et al. suggested that initial combinatorial use of antiviral is efficient in reducing short-term fatality in HBV-related ACLF (118).

The Asian Pacific Association for the Study of Liver Disease guidelines defines the importance of early administration of antiviral therapy in HBV-related ACLF and suggests that those patients with CHB, who need to undergo chemotherapy or immunosuppression procedures, require immediate antiviral treatment to prevent the consequences associated with HBV reactivation (119). While EASL and AASLD guidelines suggest that antiviral with a higher barrier to resistance are needed for patients in whom long-term antiviral prophylaxis is required, predominantly in patients who have higher HBV DNA levels before immunosuppressive therapy (120, 121).

### Treatment for Alcohol-Related ACLF

#### Corticosteroids to Treat Alcohol-Related ACLF

Active alcoholism and sAH are the most common precipitating events in ACLF in the Western world that contribute to alcohol-related ACLF (122). Unfortunately, alcoholic hepatitis often advances into multiorgan failure, leading to high mortality. To treat sAH, corticosteroids remain the first line of treatment; however, they might not be effective in all patients due to non-response to corticosteroids (123). Therefore, it is critically important to calculate the efficacy of steroids by Lille score after 4 or 7 days of treatment, which is based on age, total bilirubin levels, baseline creatinine, albumin levels, prothrombin time, and repeat total bilirubin levels (124, 125). Based on the Lille scores, patients can be categorized into a full responder, partial responder, and non-responder having a Lille score of ≤0.16, 0.16–0.56, and >0.56, respectively. Therefore, Lille score is vital to decide whether corticosteroid therapy needs to be continued or stopped. However, the prospect of response to corticosteroids confide in the existence and non-existence of ACLF. Undoubtedly, the corticosteroid response is lower in patients with ACLF than those without ACLF, and further decreases with ACLF grades (126), which could be due to the fact that corticosteroids are more effective in patients at the preliver failure stage. Patients who have the Lille score <0.45 designate poor response to corticosteroids and poor survival rate at 6 months (125). On the other hand, corticosteroid responders get survival benefits, but due to the risk of bacterial infection, careful evaluation considering the risk to benefit ratio should be investigated prior to the introduction of corticosteroids in ACLF and sAH patients. Incidence of bacterial infection is higher in corticosteroid non-responder than in the responder. Prednisolone, a corticosteroid with antiinflammatory action is widely recommended at a dose of 40 mg/day to treat sAH patients (127). Although short-term use of corticosteroids has promising results, showing a reduced risk of 1-month mortality, long-term use does not appear useful and does not improve survival beyond 1 month (128). Since, infections are common in alcoholic hepatitis patients and corticosteroids suppress the immune system, by reducing the proinflammatory cytokines such as tumor necrosis factor-α, and increases antiinflammatory cytokines, including IL-10 to reduce inflammation, immune suppression mediated by corticosteroids will further increase the incidence of bacterial infection in these patients; therefore, long-term use of corticosteroids should be avoided to lessen the risk of bacterial infection. Furthermore, application of corticosteroid therapy imposes the risk of sepsis development; therefore, selection of patients for corticosteroid treatment is critical.

#### Pentoxifylline for the Treatment of Alcohol-Related ACLF

In addition to corticosteroids, Pentoxifylline (PTX) is also considered for the treatment of alcoholic hepatitis (127). PTX is a non-phosphodiesterase inhibitor that possesses antiinflammatory properties, inhibits TNF-α production, and has anti-fibrogenic properties (129). The useful effects of PTX are also related to the downregulation of IL-1, IL-6, transforming growth factor-beta (TGF-β), interferon gamma (IFN-γ), inhibition of stellate cell activation, and procollagen I messenger ribonucleic acid expression in rats (130). Moreover, it can efficiently decrease the risk of Hepatorenal Syndrome (HRS) (131). Serum TNF-α levels are elevated in ALD, especially in alcoholic hepatitis, and the use of infliximab and etanercept, TNF-α inhibitors, increased mortality in these patients; therefore, PTX appeared useful in preventing HRS in sAH patients.

Both Prednisolone and PTX are beneficial in treating sAH; however, PTX is possibly superior to prednisolone in the cases where contradictions exist for the use of corticosteroids. Studies also investigated the use of combination therapy of Prednisolone and PTX to treat sAH patients; however, the results did not show any additional benefits in terms of morbidity and mortality (132). While treatment of patients with sAH with PTX provides promising results, it is not recommended as a first line of treatment due to the lack of evidence for its efficacy in comparison to the standard treatment with corticosteroids. However, the American Association for the Study of Liver Disease guidelines recommended the use of PTX for sAH, particularly, when contradictions exist for the use of corticosteroids (133). Also, the European Association for the Study of Liver guidelines recommended the use of PTX for the cases where the presence of sepsis inhibits the use of corticosteroids (134).

#### N-Acetylcysteine for the Treatment of sAH

The underlying molecular mechanisms of alcoholic liver disease pathogenesis are multifaceted and have not been completely elucidated. However, oxidative stress has been shown to play a critical role in mediating the inflammatory response and causing liver damage (55). Therefore, a therapeutic strategy that could control or prevent oxidative stress might be helpful in patients with ALD. N-acetylcysteine (NAC) is an antioxidant that neutralizes free radicals by increasing the intracellular glutathione and counteracting oxidative stress and inflammation protecting cells from damage (135). In case of the liver, NAC protects against liver injury by restoring hepatic glutathione supplies (136). Hence, NAC might serve as an option for the treatment of sAH patients. Several studies investigated the efficacy of NAC for the treatment of ALD and inconsistent findings were revealed. A study by Nguyen-Khac et al. discovered that intravenous administration of NAC in combination with prednisolone may be beneficial for the patients with alcoholic hepatitis. The study reported that the combination of NAC + prednisolone improved 1-month survival in comparison with those patients who only received prednisolone. However, the combination of NAC + prednisolone did not improve 6-month survival (137). Also, recent studies confirmed no 90-day survival benefit of the combination use NAC + prednisolone over prednisolone alone (138) and combination of G-CSF + NAC and G-CSF alone (139). In fact, a high dose of intravenous NAC for 14 days along with enough nutritional support neither delivered any survival benefits nor early biological improvement in sAH. MELD score and bilirubin level also did not improve at 7 and 30 days and the trials were terminated due to futility (140).

### Antimicrobial Therapies for the Treatment of Bacterial and Fungal Infections

A high percentage of patients with ACLF encounter infections either as a main precipitating event or as one of the complications in ACLF. In fact, one-third of the ACLF cases are infected with multidrug resistance (MDR) pathogens; however, that varies according to the region (100, 141). A broad spectrum of antibiotics is required if the infection is severe or MDR pathogens are present (49). Since a majority of the patients with ACLF reveal infection sooner or later, complete screening of infections is beneficial for early detection and quick antibiotic treatment. If CRP or PCT, predictors of infection, are positive, treatment should be given immediately. The selection of preliminary treatment is extremely important as it governs the patient's outcome. In the absence of suitable antibiotics, the risk of mortality increases to 74% (49). Also, therapeutic interventions primarily depend on the type, severity, and site of infection. Infections in ACLF could be community-acquired, healthcare-acquired, or nosocomial. If the infection is acquired through the community, third-generation cephalosporins are beneficial. In case of nosocomial infections, the selection of antibiotics with a broader antibacterial spectrum such as carbapenems is recommended. However, there is no standardized therapy for cirrhotic patients with nosocomial infection, as the local resistance spectrum needs to be considered. A randomized study for hospital-acquired SBP infection investigated the effect of Ceftazidime with Meropenem plus Daptomycin treatment and found that Meropenem plus Daptomycin enhanced the response to 86%, which was initially 25% (142). Further, it increased the probability of survival by 94% compared with 50% non-responders. Guidelines acclaim the use of Piperacillin/Compactum or Meropenem plus glycopeptides in nosocomial infections (143). Patients infected with SBP also require albumin replacement therapy to prevent HRS and improve outcomes (49, 144). Also, the presence of Gram-positive bacteria in cellulitis and soft tissue infections reveal the requirement of adding Oxacillin or glycopeptides. Regardless of suitable antibiotic treatment, bacterial infections display poor outcomes leading to worse clinical courses, high admissions in ICU, and short-term mortality in ACLF. Non-response to antibiotics might be due to either bacterial resistance or fungal infection requiring further investigations for advancement in the treatment strategies. Although the main cause of infection in cirrhotic patients remains bacterial infection, a fungal infection also accounts for 2–4% of the patients and causes serious complications and mortality increased by up to 70% (112). In the case of fungal infection, antimycotic therapy needs to be administered (145). Currently, there is no evidence suggesting the association of fungal infection with acute deterioration of previously compensated or decompensated cirrhosis. Patients with fungal infection are expected to have anemia, elevated bilirubin, and alkaline phosphatase in comparison with Gram positive and negative bacterial infections (112). Elevated MELD score closely associates with fungal infection. Patients with fungal infection require longer hospitalization, frequent readmissions, and are at higher risk of death.

Fungal infections particularly, invasive pulmonary aspergillosis (IPA), are detected in patients with sAH, decompensated cirrhosis, liver failure, and ACLF (146). IPA infection is prevalent in patients with ACLF and reported in 5–8.3% of HBV-related patients with ACLF and reaching ~14% in patients with sAH (147, 148). The short-term mortality is extremely high 73.5–100% (149, 150), although, at present, there are no criteria available to identify the patients with poor prognosis. Prescription drugs for the treatment of IPA include voriconazole, liposomal amphotericin B, and echinocandins, of which voriconazole is recommended as the first-line option for primary treatment of IPA (151); though due to the hepatotoxic nature of voriconazole and absence of pharmacokinetics or pharmacodynamics its use is limited in patients with ACLF (152); therefore the optimization of the voriconazole treatment regimen is strongly needed. A study tried to establish an optimal voriconazole regimen in patients with ACLF using a therapeutic drug monitoring method (153). Based on plasma voriconazole concentration measurement, an optimal voriconazole regimen including loading doses: 0.2 g twice daily and maintenance doses 0.1 g once daily was established. It was found that the voriconazole regimen was able to maintain stable and rational therapeutic trough concentrations ranging from 1 to 5 μg/mL, and patients treated with optimal voriconazole regimen displayed good clinical outcomes and higher 90-day survival i.e., 75% who also correspond to early IPA diagnosis as designated by lower CLIF-SOFA lung score (<2) in all patients preceding optimal regimen prescription. CLIF-SOFA lung score >1 was able to identify patients with ACLF complicated with IPA, encountering a much higher 28-day mortality. Also, the optimal voriconazole regimen appeared to be safe and did not display any adverse events.

### Stem-Cell-Based Therapies

**Figure 4:**
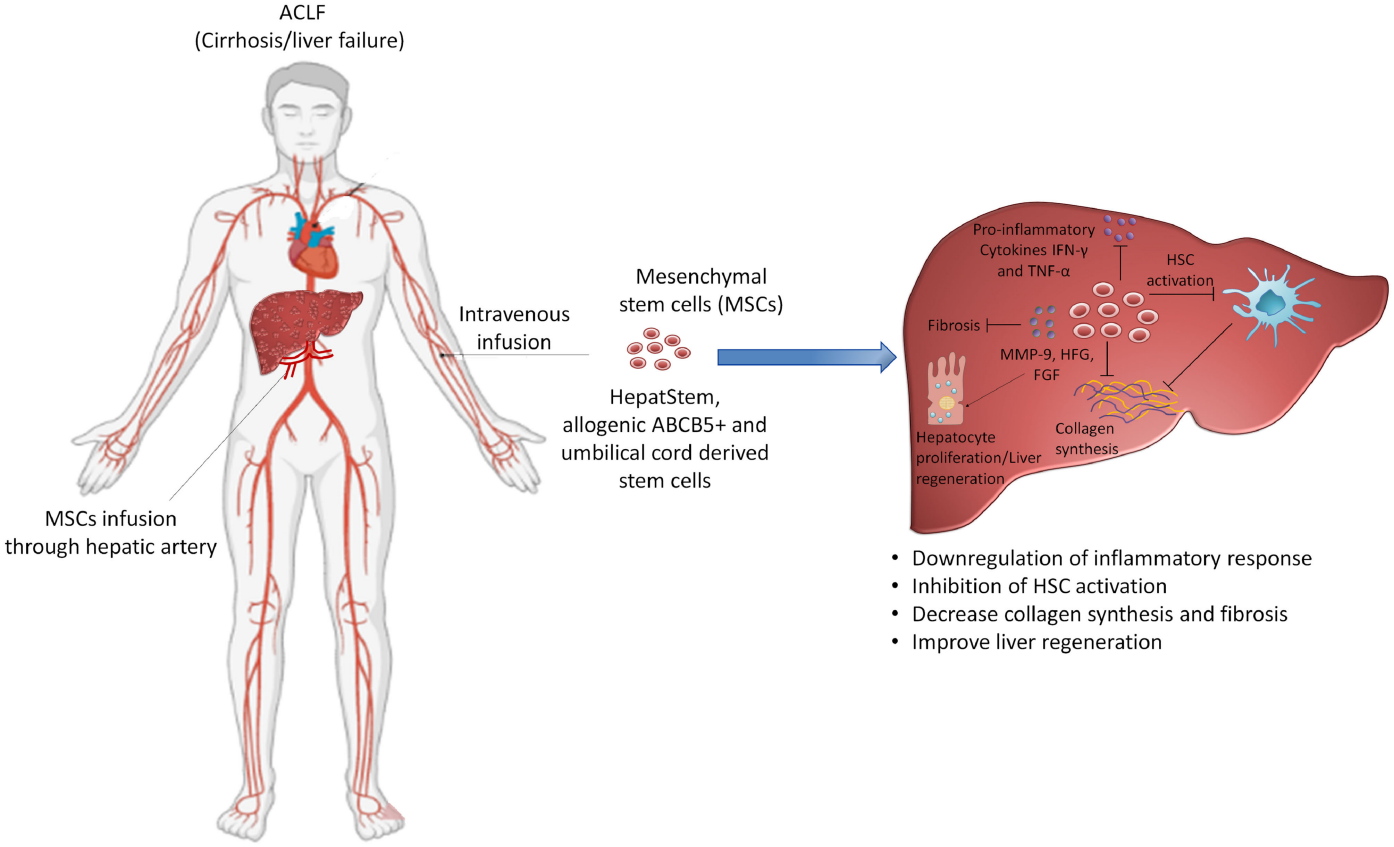
Targeting stem cell therapy for the treatment of ACLF. Use of mesenchymal stem cells is under consideration and is being investigated in clinical trials for the treatment of ACLF. Mesenchymal stem cells possess different functions including the suppression of hyperactive inflammatory response, inhibition of hepatic stellate cell activation and collagen synthesis, reducing fibrosis and inducing liver regeneration. The diverse role of mesenchymal stem cells may benefit patients with ACLF and emerge as a potential therapeutic option for these patients. HSC: hepatic stellate cells. Some parts of this figure (Human structure) were prepared with the help of BioRender.

Stem cell technology has provided hope to identify new expandable sources that can induce liver regeneration. Recently stem cell-based therapies are getting enough attention for the treatment of patients with ACLF (154–157). MSCs have massive expansion potential in the culture system and play a crucial role in tissue repair and regeneration by differentiating into several cell types and replacing the injured tissues (158). The homing potential of MSCs to the site of injury extended the spectrum of therapeutic application including the models of hepatic injury (159). After homing into the liver MSCs transdifferentiate into hepatocytes in the local microenvironment and improve hepatocyte damage and promote liver regeneration. There are ongoing phase II clinical trials (NCT04229901, NCT02946554) investigating the efficacy of HepaStem cells, a highly advanced cell therapy platform comprising human-liver-derived MSCs obtained from healthy donors and expanded in the lab. After intravenous administration, HepaStem cells migrate to the liver through circulation where they perform various functions including the downregulation of proinflammatory response, inhibition of hepatic stellate cell (HSC) activation, and reduction of collagen secretion, ultimately reducing fibrosis. An imbalanced extracellular matrix synthesis and degradation mediated by portal fibroblasts, bone marrow-derived fibroblasts, and activated hepatic stellate cells initiate hepatic fibrosis in ACLF. Immunomodulatory as well as antifibrotic function of HepaStem cell might be beneficial for treating patients with ACLF (156). MSCs mediate their antifibrotic function by downregulating myofibroblasts, which leads to antifibrotic activity (160). MSCs secrete several growth factors which stimulate resident cells and induce matrix remodeling to promote the differentiation of native progenitor cells and initiate the recovery of injured cells (160). In addition, they also possess antioxidant properties and cytoprotective effects by inducing antioxidant response elements in CCL4 and thioacetamide-induced liver injury (161, 162). Umbilical-cord-derived MSCs and also allogenic ABCB5-positive MSCs that improve liver fibrosis enhance regeneration, suppress inflammation, and downregulate Notch and Stat1/Stat3 signaling in rats (163) are under clinical trials (NCT04822922, NCT03860155) for the treatment of patients with ACLF. Although the mechanisms of MSCs have been well-described in CLDs, the mechanistic approach of MSCs in the treatment of ACLF is not well-documented since it was recently introduced as a therapeutic intervention for ACLF and clinical trials are ongoing. It is believed that immunomodulatory and antiinflammatory function of MSCs relieve hepatic inflammation, improve liver function, decrease the incidence of infection, and enhance survival rate as shown in a prospective randomized controlled clinical trial which investigated the safety, efficacy, and outcome of MSCs in HBV-related ACLF after intravenous infusion (164). The study reported that there were no infusion-related side effects except more frequent fever than patients who received standard medical therapy. Clinical laboratory measurements including total bilirubin and model for end-stage liver disease scores were improved and the incidence of severe infections was decreased (164). In fact, multiorgan failure and severe infection-related mortality were significantly lower in the MSC group. Importantly, the 24-weeks survival rate of the MSC group was higher (73.2%) than the standard medical treatment patients' group (55.6%). Another study also examined the long-term efficacy of autologous bone marrow mononuclear cells (BM-MNCs) transplantation through the hepatic artery and checked the improvement in terms of hepatic functions and decreasing complications in patients with decompensated cirrhosis (165). The study reported that the efficacy of BM-MNCs transplantation persisted for 3–12 months in comparison with the control group. Serious complications including HE and SBP were declined significantly; however, these improvements vanished after 24 months of transplantation. Few other clinical trials also demonstrated the beneficial role of MSCs in cirrhosis and patients with ACLF; therefore, regenerative medicine using stem cell technology appears promising to treat patients with ACLF and may help in reducing the requirement of liver transplantation (Figure 4).

### Granulocyte-Colony Stimulating Factor Therapy

Granulocyte colony stimulating factor (G-CSF) is a glycoprotein, which stimulates the bone marrow production of stem cells and granulocytes and releases them into the circulation. Several clinical trials studied the efficacy of G-CSF for the treatment of patients with ACLF. In fact, our previous studies identified the role of G-CSF therapy in patients with ACLF. One week of G-CSF treatment increased leukocyte as well as neutrophil count and reduced disease severity indices in patients with ACLF. G-CSF therapy also prevented the development of sepsis, HRS and HE, and improved survival of these patients (166). CD34 expressing cells are generally considered to be hematopoietic stem cells that differentiate into all hepatic cell types and recover hepatic damage by inducing liver regeneration. We observed an increase in the mobilization of CD34+ stem cells after G-CSF treatment. Moreover, G-CSF has immunomodulatory effects shown by an increase in myeloid dendritic cells and a decrease in IFN-γ producing T cells after G-CSF therapy in patients with ACLF, which is beneficial in terms of reducing IFN-γ mediated inflammation and hepatic damage in these patients (32). G-CSF therapy may also benefit patients with alcoholic hepatitis, considering that these patients are at increased risk of developing bacterial infections. Our group has previously shown that G-CSF in combination with erythropoietin can decrease the risk of septic shock in patients with decompensated cirrhosis (167). On the contrary, a recent European multicenter clinical trial reveals that G-CSF does not have any superior benefits than the standard medical treatment. The findings unveil that G-CSF is ineffective in improving patient survival and other clinical endpoints including MELD score, CLIF-C organ failure score, and the occurrence of infection, recommending G-SCF not be used as a standard treatment for ACLF (168).

### Liver Transplantation

Liver transplantation remains the ultimate therapy for patients with ACLF who face unsuccessful medical treatment (169). However, it is not feasible in all patients because of its high cost and lack of liver donors. Since, liver transplantation may be a lifesaving treatment, critical evaluation of final indication of liver transplant such as high MELD score, complications due to cirrhosis such including ascites, variceal hemorrhage, and HE should be considered (170). Patients with high MELD score have quick access to liver transplant; though, the requirement of suitable organ donors impose a major limitation (171). Liver transplant in severely ill patients with cirrhosis and organ failures is fetching attention and performed more frequently. Patients with ACLF with grade 1 and 2 display a similar posttransplant survival rate as those without ACLF (172). One-year post transplant survival rate of patients with ACLF with grade 3 varies between 44 and 83%. Since ACLF patient has poor short-term prognosis, a liver transplant might be a suitable therapeutic strategy for these patients; though the prioritization for liver transplant in these patients is quite challenging. It is important to consider if the patient has enough reserve to survive the preoperative and operative period and will have significant survival and quality of life from the liver transplant. Another major issue with the liver transplant is to choose the timing. Some recommend prioritizing the liver transplant of patients with ACLF in the waiting list after an initial stabilization. However, liver transplant in alcoholic hepatitis remains controversial as these patients drink often until or right before their presentation (173). Alcohol abstinence for 6 months is the universal requirement for the evaluation of liver transplant in these patients (174). Moreover, the shortage of liver donors along with the concerns for relapse, as it is a self-indulgent disease, enforce constant challenges to consider liver transplant as a therapeutic option for patients with sAH. The rule for 6-month alcohol abstinence is to allow the liver to get enough time to improve and regenerate. If this period does not improve liver function and/or decrease episodes of decompensation, a liver transplant can be considered. However, the concept of 6-month alcohol abstinence was challenged by a study where patients with sAH underwent liver transplant due to the steroid non-response (175, 176). Six-month survival of these patients was greatly improved as compared with those who did not receive a liver transplant, suggesting the requirement of early liver transplantation in patients with sAH (177, 178). Table 1 illustrates different ongoing clinical trials for the treatment of patients with ACLF.

**Table 1:** Clinical trials for the treatment of ACLF.

**Drug/Therapy**	**Target**	**Phase**	**Trial number**
HepaStem	Inhibits HSC activation, reduce collagen secretion and downregulation of pro-inflammatory environment.	II	NCT04229901
HepaStem	Mentioned above	II	NCT02946554
Umbilical cord derived MSCs	Improves liver fibrosis and regeneration	II	NCT04822922
Allogenic ABCB5-positive MSCs	Suppress inflammation and improve wound healing	I/II	NCT03860155
Combination of Simvastatin plus Rifaximin	Simvastatin reduce HSC activation and proliferation, increase liver sinusoidal function and decrease inflammation, Rifaximin prevent hepatic encephalopathy	IIIII	NCT03780673
Ribavirin	Hepatitis E virus infection	II	NCT01698723
PEG3350	Hepatic encephalopathy	IV	NCT03987893
Branched Chain Amino Acids	Hepatic encephalopathy	I	NCT04238416
Thymosin-α1	Treats immune suppression	N/A	NCT03082885
RL-1 Novel Human-derived bio artificial liver treatment	Improves hepatic function	N/A	NCT04195282
Albumin Plus Midodrine vs. Albumin	Reduces incidence of paracentesis induced circulatory dysfunctions	N/A	NCT04474262
Glucocorticoids	Inhibits inflammation	N/A	NCT01344174

*HSC, Hepatic stellate cells; MSCs, Mesenchymal stem cells; N/A, Not applicable*.

## Conclusion

Acute-on-chronic liver failure is a distinct entity that differs from decompensated cirrhosis in terms of clinical presentation, pathophysiology, and prognosis. Despite the progress in the understanding of pathophysiological mechanisms in ACLF, there is no specific treatment available for these patients. Also, ACLF develops as a consequence of underlying CLD, compensated, and decompensated cirrhosis; hence, it is difficult for the clinicians to identify this syndrome at the initial stage, limiting the chances of recovery. Patients with ACLF have numerous complications that require separate diagnosis and treatment. Moreover, heterogeneity in the definition of ACLF in the Eastern and Western world restricts from exact characterization and universally accepted definition, which may also lead to differences in therapeutic approaches. Liver transplantation is unanimously accepted and the only definitive therapy for these patients in the Eastern and Western world, irrespective of different ACLF definitions and regional disparity.

Recently, stem cell technology has provided new hope to identify expandable sources that can induce liver regeneration in patients with ACLF. Ongoing clinical trials are investigating the efficacy of MSCs for the treatment of ACLF as they can reduce the inflammatory response, HSC activation, collagen secretion, and fibrosis, thereby improving liver regeneration. If the approach of regenerative medicine succeeds in the field of ACLF, it will be a milestone in providing new treatment options. Since, systemic inflammation is a major contributory factor in the worsening of ACLF condition, therapeutic approaches specifically targeting excessive inflammation are also warranted for better outcomes.

## Author Contributions

AK: conceptualized, drafted, edited the manuscript, and prepared the illustrations. SK: reviewed and edited the manuscript. Both authors approved the submitted version of the manuscript.

## Conflict of Interest

The authors declare that the research was conducted in the absence of any commercial or financial relationships that could be construed as a potential conflict of interest.

## Publisher's Note

All claims expressed in this article are solely those of the authors and do not necessarily represent those of their affiliated organizations, or those of the publisher, the editors and the reviewers. Any product that may be evaluated in this article, or claim that may be made by its manufacturer, is not guaranteed or endorsed by the publisher.
